# Supplementary material for: Willingness to pay for improvements in rural sanitation: Evidence from a cross-sectional survey of three rural counties in Kenya
Source: PLoS One. 2021 May 3;16(5):e0248223. doi: 10.1371/journal.pone.0248223 (PMC8092787; doi:10.1371/journal.pone.0248223)
Supplement: S1 Appendix — (PDF) [file pone.0248223.s001.pdf]

S1 Appendix. **Survey tools administered to respondents (pdf)**

Willingness to Pay (WTP) Contingent Valuation Method (CVM) Household questionnaire;

Questionnaire code___________________ Name of interviewer_____________________ Date________________

County name__________________________

**Section 1: Household characteristics**		
1.	Respondent code;	___________________________
2.	Gender	1. Male 2. Female
3	Marital Status	1. married<br>2. single<br>3. window/widower<br>4. divorced<br>5. separated
4	Age of Household Head	_________________
5.	Is the respondent the head of the household?	1. Yes >>Q7 2. No>>Q6
6.	If No. What is the relationship with the Head of household?	1. wife<br>2. daughter/Son<br>3. grandmother/father<br>4. daughter/ son in law<br>5. others specify____________________
7	How many people live in this household?	__________________
8.	How many are adults over 18 years?	__________________
9	How many elderly people aged 60 yrs and above are there in the	__________________

	household?	
10	How many are young people aged (13-18)	____________________
11	How many children under the age of 5yrs are there in your household?	____________________
12	How many rooms in this household are used for sleeping?	__________________________
13	What is the highest level of education completed by the head of household?	1. Primary<br>2. Post-Primary/Vocational<br>3. Secondary/'A' Level<br>4. College (Middle Level<br>2. University
14	What is the current employment/occupation of the household head?	1. Never had a job<br>2. subsistence farmer<br>3. commercial farmer<br>4. farm worker<br>5. Fisherman<br>6. Trader/hawker/vender<br>7. artisan<br>8. government worker<br>9. housewife<br>10. student<br>11. Others specify_____________________
15	What is your Estimated Monthly Income?	Ksh___________________________
16	What is the main material the	1. Earth/Sand<br>2. Dung

	floor your house is made of?	3. Wood Plank<br>4. Palm/Bamboo<br>5. Paraquet or Polished wood<br>6. PVC/Vinyl or Asphalt Strips<br>7. Ceramic Tiles<br>9. Cement<br>10. Carpet<br>11. others specify________________
17	What is the main material the walls of your house are made of?	1. No walls<br>2. Cane/Palm/Trunk<br>3. Dung/mud<br>4. Bamboo with mud<br>5. Stone with mud<br>6. Uncovered adobe<br>7. Plywood<br>8. Cardboard<br>9. Reused wood<br>10. Mabati/iron sheet<br>11. Cement<br>12. Stone with lime/cement<br>13. Bricks<br>14. Cement block<br>15. Covered adobe<br>16. Wood planks/shingles<br>17. others specify__________________
18	What is the main material the roof of your house is made of?	1. No roof<br>2. Thatch/grass/makuti<br>3. Dung/mud/<br>4. Iron sheets/Mabati<br>5. Tin Cans<br>6. Asbestos sheet<br>7. Concrete<br>8. Tiles

		9. others specify
19	Which one of the following items do you own in your house?	Yes No<br>Electricity 1 2<br>Radio 1 2<br>Television 1 2<br>Mobile phone 1 2<br>Non-mobile phone 1 2<br>Computer/laptop 1 2<br>Refrigerator 1 2<br>Solar panel 1 2<br>Table 1 2<br>Chairs 1 2<br>Sofa set 1 2<br>Bed 1 2<br>Cupboard 1 2<br>Clock 1 2<br>Microwave Oven 1 2<br>DVD Player 1 2<br>CD player 1 2<br>watch 1 2<br>Bicycle 1 2<br>Motorcycle 1 2<br>Car/track 1 2<br>Ox-drawn plough 1 2<br>Boat with motor 1 2<br>Others Specify______________________
**Section 2: Household Water, Hygiene and sanitation**		
S1	What kind of toilet facility do members of your household usually use? *If "flush" or "pour flush" probe:* Where does it flush to? ________________________	1 Flush/pour flush<br>2 Ventilated improved pit latrine (VIP)<br>3 Pit latrine with slab

		4 Pit latrine without slab/open pit<br>5 Composting toilet<br>6 Bucket<br>7 Hanging toilet/hanging latrine<br>8 No facilities or bush or field<br>9 Other specify__________________
S2	Do you share this facility with other households?	1. Yes 2. No. >>S4
	How far away from your house is the toilet facility located?	________________________
S3	How many households use this toilet facility?	________________________
S4	How many other households share this toilet?	_______________________
S5	Can any member of the public use this toilet?	1. Yes 2. No. 3. DK
S6	How satisfied are you with your current toilet facility?	1. Very satisfied 2. Satisfied<br>3. moderately 4 not satisfied
S7	Do you believe that unsanitary toilets, leads to diseases?	1. Yes 2. No. DK

S8	Would you be interested in improving your current toilet facility if you are able do so?	1. Yes 2. No. >>S6 DK
S9	What is the main source of water used by your household for domestic purposes, such as cooking and hand washing?	1 Piped water into dwelling<br>2 Piped water to yard/plot<br>3 Public tap/standpipe<br>4 Tube well/borehole<br>5 Protected dug well<br>6 Unprotected dug well<br>7 Protected spring<br>8 Rainwater collection<br>9 Surface water (river, dam, lake, Pond, stream)<br>10 Others Specify______
S10	What is the main source of drinking-water for members of your household?	1 Piped water into dwelling<br>2 Piped water to yard/plot<br>3 Public tap/standpipe<br>4 Tube well/borehole<br>5 Protected dug well<br>6 Unprotected dug well<br>7 Protected spring

		8 Rainwater collection<br>9 Surface water (river, dam, lake, Pond, stream)<br>10 Others Specify_______________
S11	Do you pay water for **use** in your household?	1. Yes 2. No. DK
S12	How long does it take to go there, get water, and come back?	No. of minute______________<br>1. Water on premises<br>2. DK
**Household health.**		
H1	Does anyone in your household < 5 years of age had unusual diarrheal symptoms(watery/bloody diarrheal) in the past 4 weeks	1. Yes 2. No. 3. DK
H2	Does anyone in your household > 5 years of age had unusual diarrheal symptoms(watery/bloody diarrheal) in the past 4 weeks	1. Yes 2. No. 3. DK

**Section 3 : Household Willingness to Pay (WTP) Valuations**		
**SAFI Latrine;**<br>Scenario description: USAID/KIWASH thinks that your household needs to have an improved toilet facility. This would be very good for the health of persons in your household and the environment and you as the head of the household has to pay for its construction (**show the pictures or explain if need be**). It is going to be the responsibility of the organization to set the amount people will have to pay for the construction of the toilet. Remember this has nothing to do with this survey. Would you be willing and able to pay the following costs for the stated benefits for all persons in your household?<br>1. Yes 2. No.		
**Part A**	Suppose the cost of constructing a model SAFI latrine is Ksh. 35,000, would you or someone in your household be willing and able to pay this amount?	1. Yes go to B<br>2. No go to C<br>3. Not sure go to C3
**Part B**		
B1	Enumerator repeats question but with price and card of Ksh. 25, 000	1. Yes. FINISH<br>2. No. go to B2<br>3. Not sure, go C3
B2	Enumerator repeats question but with price and card of Ksh.23, 000	1. Yes FINISH<br>2. No go to B3<br>3. Not sure C3
B3	Enumerator repeats question but with price and card of Ksh.	1. Yes. FINISH<br>2. No. go to B4

	20,000	3. Not sure, go C3
B4	Enumerator repeats question but with price and card of Ksh.18, 000	1. Yes FINISH<br>2. No go to C1<br>3. Not sure go to C3
**Part C**		
C1	Enumerator repeats question with price and card of Ksh. 12, 000	1. Yes FINISH<br>2. No go to C2<br>3. Not sure go to C3
C2	Enumerator repeats question but with price and card of Ksh.10, 000.	1. Yes FINISH<br>2. No go to C3<br>3. Not sure go to C3
C3	What is the maximum amount you would be willing and able to pay for the construction of a model SAFI latrine?	KSH______________________

**SATO Products;**<br><br>Scenario description: USAID/KIWASH thinks that your household needs to have an improved toilet facility. This would be very good for the health of persons in your household and the environment and you as the head of the household has to pay for the installation of a pan and stool slab (show the picture or explain if need be). It is going to be the responsibility of the organization to set the amount people will have to pay for the installation of a pan and stool. Remember this has nothing to do with this survey. Would you be willing and able to pay the following costs for the stated benefits for all persons in your household?		
**Part A**	Suppose the cost for installation of a pan and stool slab is set at Ksh. 1,500 would you or someone in your household be willing and able to pay this amount?	1. Yes go to B<br>2. No go to C<br>3. Not sure go to C
**Part B**		
B1	Enumerator repeats question but with price and card of Ksh. 2,500	1. Yes FINISH<br>2. No go to B2<br>3. Not sure go to B2
B2	Enumerator repeats question but with price and card of Ksh.2,300	1. Yes FINISH<br>2. go to B3<br>3. Not sure, go to B3
B3	Enumerator repeats question but with price and card of Ksh. 2,000	1. Yes FINISH<br>2. No go to B4<br>3. Not sure, go to B4

B4	Enumerator repeats question but with price and card of Ksh.1,800	1. Yes FINISH<br>2. No go to C1<br>3. Not sure, go to C4
**Part C**		
C1.	Enumerator repeats question with price and card of Ksh. 1,200	1. Yes FINISH<br>2. No. go to C2<br>3. Not sure, go to C2
C2	Enumerator repeats question but with price and card of Ksh.1, 000.	1. Yes FINISH<br>2. No go to C3<br>3. Not sure, go to C3
C3	Enumerator repeats question but with price and card of Ksh. 800	1. Yes FINISH<br>2. No go to C4<br>3. Not sure, go to C4
C4	What is the maximum amount you would be willing and able to pay for the installation of a pan and stool slab?	Ksh. ___________________
**Section 4 : Role of credit on WTP for SAFI latrine**		

Now the organization would like to offer you an in- kind loan to help you acquire a SAFI toilet facility, for which you will pay a 10% interest rate per month on a reducing balance basis for a period of 12 months. You will be expected to make monthly repayment until the loan amount is cleared (Capital +interest). **The numerators should take at least 5 minutes to explain to the respondents about the loan facility terms very clearly, Before asking the following questions.**

D1	Are you a member of any community group or organization?	1. Yes >>D2<br>2. No

D2	If YES, what kind of organization are you a member of?	1. Community based organization (CBO)<br>2. Faith Based Organization (FBO)<br>3. Non-Government Organization (NGO)<br>4. Business Community<br>5. Others (SPECIFY)___________
D3	Have you taken a loan in the past one year	1.Yes >>D4<br>2. No
D4	If, YES, what organization financed you with the loan?	1. Microfinance<br>2. Commercial bank<br>3. Government<br>4. Village Saving and Loan groups (VSLA)<br>5. SACCO<br>6. Cooperative<br>7. Friends/Relative
D5	Would you or any member of your household accept the in-kind loan for the construction of a SAFI Toilet, if it's offered to you?	1. Yes<br>2. No.
D6	Has any member of your household received any free gifts/subsidies from the Government or other institutions	1. Yes<br>2. No

	in the last two years?	

Thank the respondent and leave for the next households.
