# Supplementary material for: Willingness to pay for improvements in rural sanitation: Evidence from a cross-sectional survey of three rural counties in Kenya
Source: PLoS One. 2021 May 3;16(5):e0248223. doi: 10.1371/journal.pone.0248223 (PMC8092787; doi:10.1371/journal.pone.0248223)
Supplement: Attachment — Submitted filename: Reviewer attachment.docx [file pone.0248223.s003.docx]

**Background:** The introduction focused on highlighting generic problems to drawing on the peculiar problems in Kenya rural sanitation problems. The author focused very little attention dealing with the two technologies being adopted.

- Simple expression will be ideal. E.g.: Pro-rich could have been written as for the rich …

**Methods and materials:** The author failed to clearly explain what the CVM method is and why it would best capture their problem. The author also failed to explain how the CVM method was used. In addition, the author does not explain how the dichotomous choice method works and why it was used.

- The study site: Avoid repetition (from the introduction). For example, instead of providing general statistics on rural sanitation, the author should focus on statistics on the 3 counties

**Results**:

Table 1: Under the economic status, author should consider providing details on wealth quintile 1-2 as the poorest followed by quintile 3 as the average and the last 4-5 quintiles as the rich quintiles

It was not clear why the author examined the willingness to pay for SATO stools at a market price of $10 USD, which was way higher than the market price of the SATO stool. Author should clarify relevance of this information
