# Supplementary material for: Willingness to pay for improvements in rural sanitation: Evidence from a cross-sectional survey of three rural counties in Kenya
Source: PLoS One. 2021 May 3;16(5):e0248223. doi: 10.1371/journal.pone.0248223 (PMC8092787; doi:10.1371/journal.pone.0248223)
Supplement: Attachment — Submitted filename: Response to reviewers.doc [file pone.0248223.s004.doc]

Diana Mulatya

P.O Box 31503-00600, Nairobi

Email: [dianamulatya@gmail.com](mailto:dianamulatya@gmail.com)

Cell: +254720322653

6^th^, January, 2021

The Editorial Team,

PLOS ONE

Dear Sir/Madam

**RE: Revision one – response to comments on original Manuscript**.

I would like to submit the revised version 1 of our manuscript entitled “***Willingness to pay for improvements in rural sanitation: Evidence from a cross-sectional survey of three rural counties in Kenya*** to be considered for publication as an original research in PLOS ONE.

Below is the detailed response to all revisions requested for the manuscript.

1. Please ensure that your manuscript meets PLOS ONE's style requirements, including those for file naming. The PLOS ONE style templates can be found at

<https://journals.plos.org/plosone/s/file?id=wjVg/PLOSOne_formatting_sample_main_body.pdf>

and <https://journals.plos.org/plosone/s/file?id=ba62/PLOSOne_formatting_sample_title_authors_affiliations.pdf>

**This was done and the revised manuscript is now aligned to PLOSOne guidelines.**

2. Please ensure that you refer to Figure 6 in your text as, if accepted, production will need this reference to link the reader to the figure.

**This was corrected in the manuscript**

3. We note you have included tables to which you do not refer in the text of your manuscript. Please ensure that you refer to Tables 1 and 3 in your text; if accepted, production will need this reference to link the reader to the Tables.

**We have updated the content and all Tables are correctly referenced.**

4. Please upload a copy of Supporting Information S1 File which you refer to in your text on page 30.

**This has been uploaded and cited as S3 Appendix 1- survey data**

5. My funding statement is now updated to read;

The funder provided support in the form of salaries for authors [VW, JO, DMM], but did not have any additional role in the study design, data collection and analysis, decision to publish, or preparation of the manuscript, and only provided financial support in the form of authors' salaries and/or research materials. The specific roles of these authors are articulated in the author contribution section.

The competing interest statement was also revised to read;

This study was undertaken by a Kenyan based consultancy firm, here in referred to as AMREC consultants, and was conducted as part of a consultancy assignment funded by the USAID-Kenya Integrated Water, Sanitation and Hygiene project. This engagement does not alter our adherence to PLOS ONE policies on sharing data and materials.” (as detailed online in our guide for authors http://journals.plos.org/plosone/s/competing-interests).

The authors declare no competing interest.

Below is our response to the reviewer’s comments;

Reviewer #1: Methods
1. Please provide a reference to the statement “…. with a glaring 50.8% of the population using unimproved sanitation and another 13.9% practicing open defecation rates”

**This is now provided as reference # 6 on the manuscript. Sourced from Kenya national bureau of statistics, Kenya Household Budgetary Survey 2015/2016**

2. The authors state that the study was a quantitative “economic evaluation … ” This is misleading to readers. A Contingent Valuation Method is not a form of an economic evaluation but a stated preference elicitation approach. I suggest deleting the economic evaluation part.

**Thanks, this has been deleted (Page 6)**

Results
1. Rephrase the statement “Of the 54 households with diarrhea (9.78%), most of them 6.51% were children aged >5 years.” I suppose this should be the households with a person with diarrea?

**We agree, this is corrected (Page 11)**

2. The figure numbers in the text need to be aligned. For instance the authors refer to figure 2 to be showing approximately 3% of the households being willing to pay for the market price for SAFI latrines whereas Figure two indicates the Map of study counties.

**This have been corrected and figures aligned to the text updated**

Reviewer #2: 1. The conclusion focused more on the recommendation. Author should first provide summary of result from data with supporting information before making recommendations

**The conclusion has been reviewed accordingly and a summary of results from this study provided against each recommendation.**

3. Though the reviewer has expertise in the topic but is not specialized in using quantitative methodology (for example, the economic modeling used). Reviewer recommends having another reviewer with specialization in economic modelling)

**This comment was addressed by the editor**

*Other comments*

4. The dataset should be anonymized and made fully available without restrictions

**The attached data has been anonymized and will be made publicly available.**

5. Under economic status, an author should provide details on wealth quantile categories. Expound on the principal content analysis and how each cluster was developed.

**Household socioeconomic status (SES) indices were generated using multiple correspondence analyses using the following variables; occupation of household head, the primary source of drinking water, type of cooking fuel, ownership of household assets, and ownership of livestock. The households were categorized into five socioeconomic quintiles (wealth quintile)**

6. It's not clear why the author examined the WTP for SATO above the current market price of $10. Author to explain the relevance of this information.

**The author examined willingness to pay for SATO pans and stools well above the market prices of $6 and $10 dollar to provide for an upper price range above the recommended retail price, within which customers would still be willing to pay for SATO products. This allows the author to simulate for a real market scenario, where product prices rise above recommended retail price. This mostly happens when middle men and brokers gain entry into the product supply chain, keep on making commercial gains.**

We hope that our response explicitly answers all the comments raised in our first manuscript.

Thanks for your continued support

Yours Sincerely,

Diana Mutuku Mulatya-Masters Degree in Public Health, LSHTM

Sanitation and Hygiene Advisor

Development Alternatives Incorporation (DAI)
